# Disulphide Bridges of Phospholipase C of *Chlamydomonas reinhardtii* Modulates Lipid Interaction and Dimer Stability

**DOI:** 10.1371/journal.pone.0039258

**Published:** 2012-06-21

**Authors:** Mayanka Awasthi, Jyoti Batra, Suneel Kateriya

**Affiliations:** Department of Biochemistry, University of Delhi, South Campus, New Delhi, India; Centre National de la Recherche Scientifique, France

## Abstract

**Background:**

Phospholipase C (PLC) is an enzyme that plays pivotal role in a number of signaling cascades. These are active in the plasma membrane and triggers cellular responses by catalyzing the hydrolysis of membrane phospholipids and thereby generating the secondary messengers. Phosphatidylinositol-PLC (PI-PLC) specifically interacts with phosphoinositide and/or phosphoinositol and catalyzes specific cleavage of sn-3- phosphodiester bond. Several isoforms of PLC are known to form and function as dimer but very little is known about the molecular basis of the dimerization and its importance in the lipid interaction.

**Principal Findings:**

We herein report that, the disruption of disulphide bond of a novel PI-specific PLC of *C. reinhardtii* (CrPLC) can modulate its interaction affinity with a set of phospholipids and also the stability of its dimer. CrPLC was found to form a mixture of higher oligomeric states with monomer and dimer as major species. Dimer adduct of CrPLC disappeared in the presence of DTT, which suggested the involvement of disulphide bond(s) in CrPLC oligomerization. Dimer-monomer equilibrium studies with the isolated fractions of CrPLC monomer and dimer supported the involvement of covalent forces in the dimerization of CrPLC. A disulphide bridge was found to be responsible for the dimerization and Cys7 seems to be involved in the formation of the disulphide bond. This crucial disulphide bond also modulated the lipid affinity of CrPLC. Oligomers of CrPLC were also captured in *in vivo* condition. CrPLC was mainly found to be localized in the plasma membrane of the cell. The cell surface localization of CrPLC may have significant implication in the downstream regulatory function of CrPLC.

**Significance:**

This study helps in establishing the role of CrPLC (or similar proteins) in the quaternary structure of the molecule its affinities during lipid interactions.

## Introduction

Phosphoinositide (PIP3) has been shown to affect a great variety of cellular responses, including exocytosis, cytoskeleton remodeling, chemotaxis and regulation of ion channels [1], and its metabolism inside the cell is stringently controlled [2]. Phosphoinositide-specific phospholipase C (PI-PLC) is the class of enzymes that hydrolyzes the highly phosphorylated phosphatidylinositol 4, 5-bisphosphate [PI(4,5)P2], generating two intracellular products inositol 1,4,5-trisphosphate (InsP3) and diacylglycerol (DAG) [3]. This event serves as one of the earliest key steps to trigger further phosphoinositide-mediated intracellular signal transduction pathways [4]. InsP3 is a universal calcium-mobilizing secondary messenger [5] and DAG is an activator of protein kinase C [6], [7]. Both signaling molecules modulate intracellular Ca^2+^ level. Activated protein kinase C regulates a wide variety of downstream effectors [8]. PLCs are soluble and membrane associated multi-domain proteins that are classified into different isoforms like β, γ, δ, ζ, η and ε, on the basis of their primary structure and mechanism of their activation [9]. PLCs harboring single domain also exist in nature [10]. Different domains of PLC constitutes catalytic α/β barrel including X and Y region, which represents the incomplete TIM (triose phosphate isomerase) barrel fold, hydrophobic rim region, X/Y-spanning sequence (Z Region), pleckstrin homology (PH) domain, EF-hands, C2 domain and C-terminus extension [11]. These modular domains of PLCs are known to regulate lipid interaction and/or enzyme activity [12], [13], [14], [15].

Dimerization is one of the known molecular behaviours of phospholipases, which also plays a role in regulation of PLC activity. Some isoforms of PLC are known to exist and function as dimers, such as purified PLC from human platelets [16]. In 1994, Banno *et. al.* suggested that size exclusion chromatography purified PLC-β-dimers are catalytically active enzymes [17]. Crystal structure of avian PLC-β reveals that C-tail is composed of three long helices, forming a coiled-coil structure, which controls dimerization of the enzyme in an antiparallel orientation [18]. Catalytic domain of the bacterial (*Bacillus thuringiensis)* PLC is responsible for dimerization, which possesses only the catalytic XY domain of PLC and also forms a dimer [19], [20].

The role of PLC in the unicellular, biflagellate, fresh water alga, *Chlamydomonas reinhardtii* has been investigated during stress responses [21]. In 1992, Quarmby *et. al.* also demonstrated the role of PLC in biochemical pathways that couples deflagellation process, induced by the pH shock [22]. However, there is no molecular characterization of the phospholipase from *C. reinhardtii.* In this study, for the first time we report quaternary structure characterizations, and identify an important disulphide bridge that modulates dimer stability and alters lipid interaction affinity of the novel phospholipase C of *Chlamydomonas reinhardtii* (CrPLC). CrPLC possesses only catalytic (XY) and C2 domains. The quaternary structure characterization of CrPLC suggests that it exists as dimer in both *in vitro* and *in vivo* conditions. Immunolocalization studies with CrPLC antibody indicate its association with plasma membrane in the *Chlamydomonas* cells.

## Results and Discussion

### Bioinformatics Analysis of the Novel Phospholi Pase from *C. reinhardtii*

**Figure 1:**
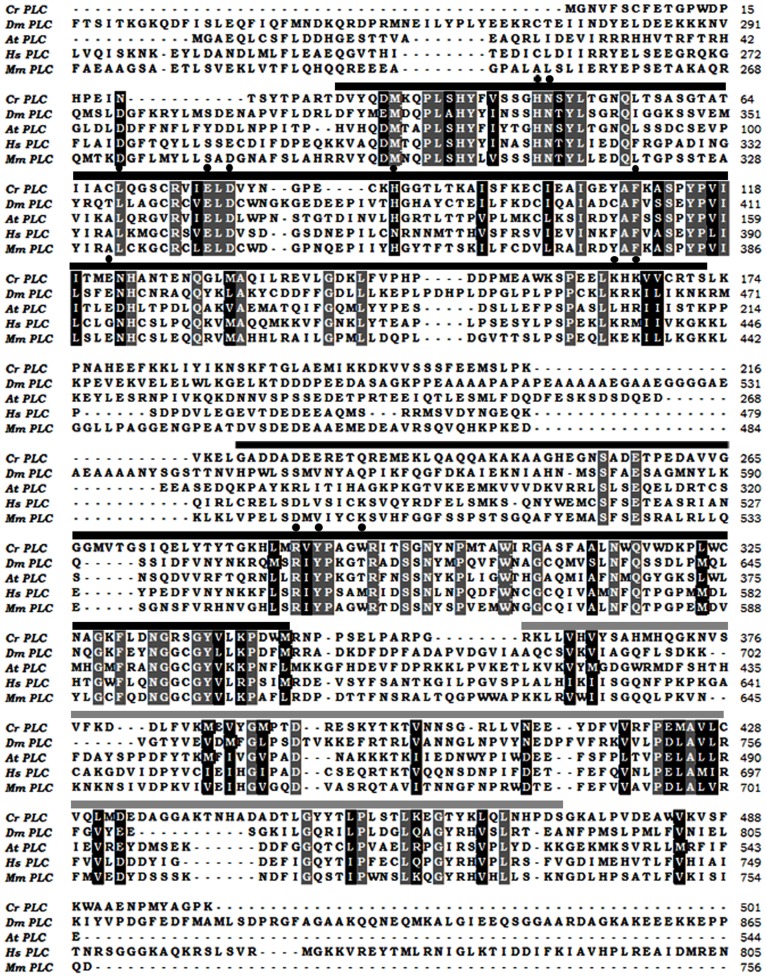
Homology analysis of PI-PLC isozymes from different organisms. Sequences of Phospholipase C from different organisms including *Chlamydomonas reinhatdtii* (CrPLC), *Drosophila melanogaster* (DmPLC), *Arabidopsis thaliana* (AtPLC), *Homo sapiens* (HsPLC), and *Mus musculus* (MmPLC) were compared. Identical amino acid residues are represented in white with black background and residues with greater than 85% similarity in sequences are highlighted in black with grey background. Residues present in the X–Y domain and catalytic domain are indicated by black and grey solid bars respectively. Catalytically important and conserved amino acid residues for substrate binding are marked by solid black circles.

Conserved domain analysis of the CrPLC protein sequence (501a.a) suggested the presence of catalytic domain and the C2 domain. Catalytic domain was found to have a significant similarity with that of the eukaryotic phosphoinositide-specific phospholipase C family, which are characterized by the presence of two well conserved histidine residues (H47 and H88) in the catalytic domain. Cluster of hydrophobic amino acid residues, which are responsible in substrate recognition, calcium binding and catalysis of PI-specific PLC [23] were also found to be conserved in CrPLC. These residues includes H47, N48, L69, E77, D79, H88, F110, E122, K164, K166, R286, Y288 and W292 in CrPLC. Domain architecture analysis of CrPLC revealed the presence of catalytic domain that constitutes conserved X and Y regions separated by a linker region and calcium binding C2 domain. Comparison of the deduced amino acid sequence of CrPLC with the other known PLC sequences showed that the conserved residues are mainly present in the catalytic domain and C2 domain (Figure 1). Phylogenetic analysis of CrPLC with the other known phospholipase suggested its close relatedness to phospholipases present in higher plants. Protein sequence alignment showed overall 45% homology with *Arabidopsis thaliana* phospholipase (Figures 1 and S1). Predicted tertiary structure of CrPLC resembled an incomplete triose phosphate isomerase (TIM) α/β-barrel structural fold. Catalytic domain in 3-D structure model exhibited alternating α-helices and β-strands whereas calcium binding C2 domain had five antiparallel β-strands arranged as a sandwich. Most of the aromatic amino acid residues including Tyr and Trp present in the tertiary structure of CrPLC was present on the surface of molecule (Figure S2). Thus, overall architecture of the tertiary structure of CrPLC would set an acceptable confirmation of the conserved aromatic rings for further molecular interactions. It could also establish strong hydrophobic interaction with residues from the other CrPLC molecule. CYSPRED server that utilizes the evolutionary information for prediction of disulphide bonding state of the cysteine, predicted that only Cys7 of CrPLC was in bonded state i.e., likely to participate in the disulphide bond formation whereas other cysteine residues were in non-bonded state (Free State). According to Plant-mPLoc analysis, CrPLC was predicted to be localized in cell membrane. This prediction suggested a possible role of CrPLC in phosphatidylinositol 4, 5-bisphosphate (PIP2) metabolism and lipid signaling pathways in the cell.

### Recombinant CrPLC Stays as Monomer and Oligomer in Solution

**Figure 2:**
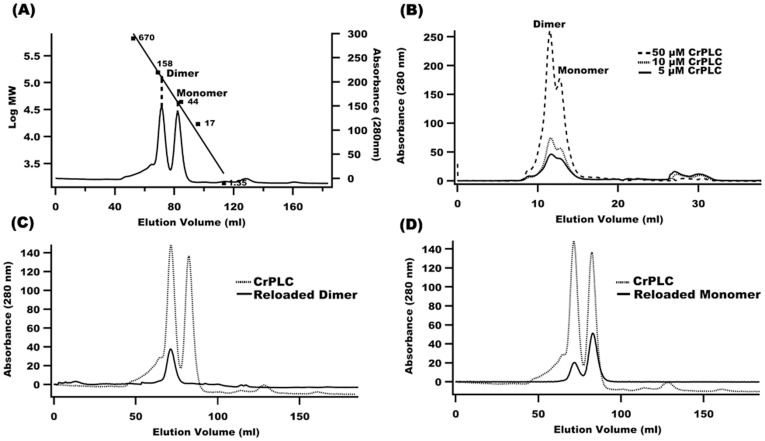
Quaternary structures of recombinant CrPLC. (A) Size exclusion chromatogram of recombinant CrPLC. Straight line depicts the calibration of standard molecular weight marker including, thyroglobulin (670 kDa), γ-globulin (158 kDa), ovalbumin (44 kDa), myoglobin (17 kDa) and vitamin B12 (1.35 kDa). (B) Size-exclusion profile of CrPLC of various concentrations. Elution profiles of CrPLC corresponding to various concentrations of recombinant CrPLC (5, 10 and 50 µM) were recorded using analytical grade size exclusion chromatography column and shown in different line patterns as mentioned in the inset of figure. (C and D) Size exclusion chromatogram of reloaded monomer and dimer fractions using preparatory grade chromatography column are shown in black, overlaid with chromatogram of CrPLC (dotted line).

The cDNA of CrPLC (1503 bp) was isolated, sequenced and nucleotide sequence encoding CrPLC deposited in GenBank (Accession number JN052078). Recombinant CrPLC was expressed mainly in soluble form and purification level was monitored by SDS-PAGE (Figure S3). CrPLC purified to homogeneity by size exclusion chromatography (SEC) indicated that it was eluted in two fractions (Figure 2A). As determined by calibration of the column with proteins of known molecular standard, fractions 22–25 (elution volume 67–76 ml) eluted at a molecular mass of 110 kDa that corresponded to the apparent molecular mass of CrPLC dimer while fractions 26–29 (elution volume 79–88 ml) corresponded to the molecular mass of CrPLC monomer (55 kDa) (Figure S4). A smaller shoulder observed towards the left of the dimer peak was attributed to the little amount of higher oligomer of CrPLC. Oligomerization in some proteins is known to be affected by dilution (concentration dependent oligomerization) [24]. However, no effect of dilution was observed on dimerization under varying concentration (5 µM to 50 µM) of CrPLC using gel filtration analytical column (Figure 2B). It has also been shown that enzyme activity of some phospholipases was modulated by reversible dimerization of enzyme [25], [26]. To assess the monomer-dimer equilibrium of CrPLC, eluted monomer and dimer fractions were reloaded on the preparatory SEC column and eluents were analyzed separately. Reloading of CrPLC monomer and dimer fractions of CrPLC resulted in slight shifting of the monomer elution peaks towards dimer fraction indicating that the purified monomer fraction of CrPLC could form dimer, however no conversion of dimer fraction into monomer was observed, this suggested the existence of a very high-energy barrier between these states (Figures 2C and D) CrPLC monomer was found to form dimer under oxidative conditions, which was not affected by protein dilution, this implied the involvement of strong covalent forces in stabilization of CrPLC dimer.

### CrPLC Dimerization is Stabilized by Disulphide Bond in Both *in vitro* and *in vivo* Conditions

**Figure 3:**
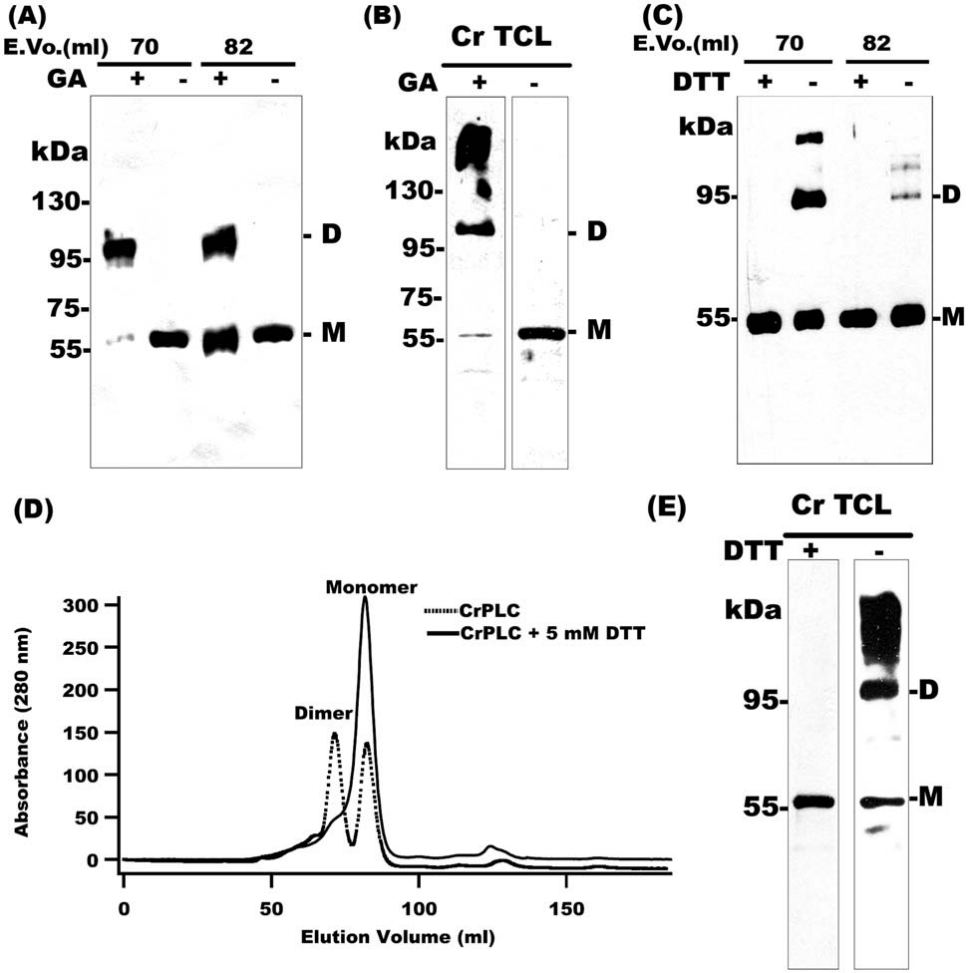
CrPLC dimerizes both *in vitro* and *in vivo*. (A) Immunoblotting of crosslinked (Glutaraldehyde; GA) dimer and monomer peak fractions. Numbers above each lane are the elution volume in ml with 70 ml (dimer peak fraction) and 82 ml (monomer peak fraction). (B) Cellular detection of CrPLC dimer and monomer species by glutaraldehyde (GA) crosslinking of *Chlamydomonas* total cell protein (Cr TCL) followed by immunoblotting. (C) Immunoblot analysis of CrPLC dimer and monomer peak fractions of size exclusion chromatography in both presence (reducing) and absence (non-reducing) of dithiothreitol (DTT). (D) Comparative chromatogram of recombinant CrPLC performed under reducing condition using 5 mM DTT (black) and in non-reducing condition (red). (E) Cellular detection of CrPLC in *Cr* TCL under reducing and non-reducing conditions. Dimer and Monomer in the blots are marked as D and M respectively.

Dimerization of recombinant CrPLC was interesting in the light of the fact that phospholipase dimers are known to form functional complexes. Previous studies suggested the role of dimerization as the molecular mechanism by which functional role of phospholipases were regulated inside the cell [25]. Chemical crosslinking of CrPLC using glutaraldehyde crosslinking agent, captured dimer of CrPLC as observed on SDS-PAGE (data not shown). Immunoblotting of chemically cross-linked monomer and dimer fractions using CrPLC specific antibody showed that the monomer fraction was present in both monomer and dimer forms, whereas the dimer fraction was found to be completely cross-linked (dimer). CrPLC dimer *in vivo* condition was also determined by similar protein crosslinking experiments. *Chlamydomonas* total cell lysate (Cr TCL) was cross-linked and CrPLC was probed using specific antibody. Interestingly, by glutaraldehyde crosslinking of Cr TCL, we were able to detect both CrPLC dimer and monomer forms. The formation of higher oligomer was also observed in *in vivo* conditions, which could be due to some degree of nonspecific crosslinking (Figure 3B). Thus, our findings suggested that CrPLC exist as dimer in both *in vitro* and *in vivo* conditions. Various non-covalent forces and covalent interactions like disulphide bridges are known to play a role in the formation and stabilization of the quaternary structure of the proteins. To explore the primary driving force involved in the stabilization of quaternary structure of CrPLC, effect of reducing and non-reducing conditions were compared simultaneously. In SDS-PAGE, CrPLC migrated predominantly as a dimer of molecular weight 110 kDa under non-reducing conditions. This higher molecular weight band totally disappeared under reducing conditions and CrPLC was detected as a monomer of 55 kDa only (data not shown). Immunoblotting of SEC purified monomer and dimer peak fractions (elution volume 70 and 82 ml respectively) under reducing and non-reducing conditions also showed the presence of dimer under non reducing conditions (Figure 3C). Small amount of higher oligomers were also detected by immunoblotting under non reducing condition (Figure 3C). Moreover, size exclusion chromatography with 5 mM DTT in the elution buffer resulted in elimination of the dimer peak (Figure 3D). To find out the involvement of disulphide bond(s) in native CrPLC, CrPLC was probed in the *Chlamydomonas* total cell protein by immunoblotting under both reducing and non-reducing conditions. Similar to that of recombinant CrPLC, under non-reducing conditions, both monomer and dimer were detected, whereas in the presence of DTT, dimer completely disappeared (Figure 3E). Thus, disulphide bonds were found to contribute significantly in CrPLC dimerization in the native protein as well.

### Cys 7 Plays Key Role in CrPLC Dimerization

**Figure 4:**
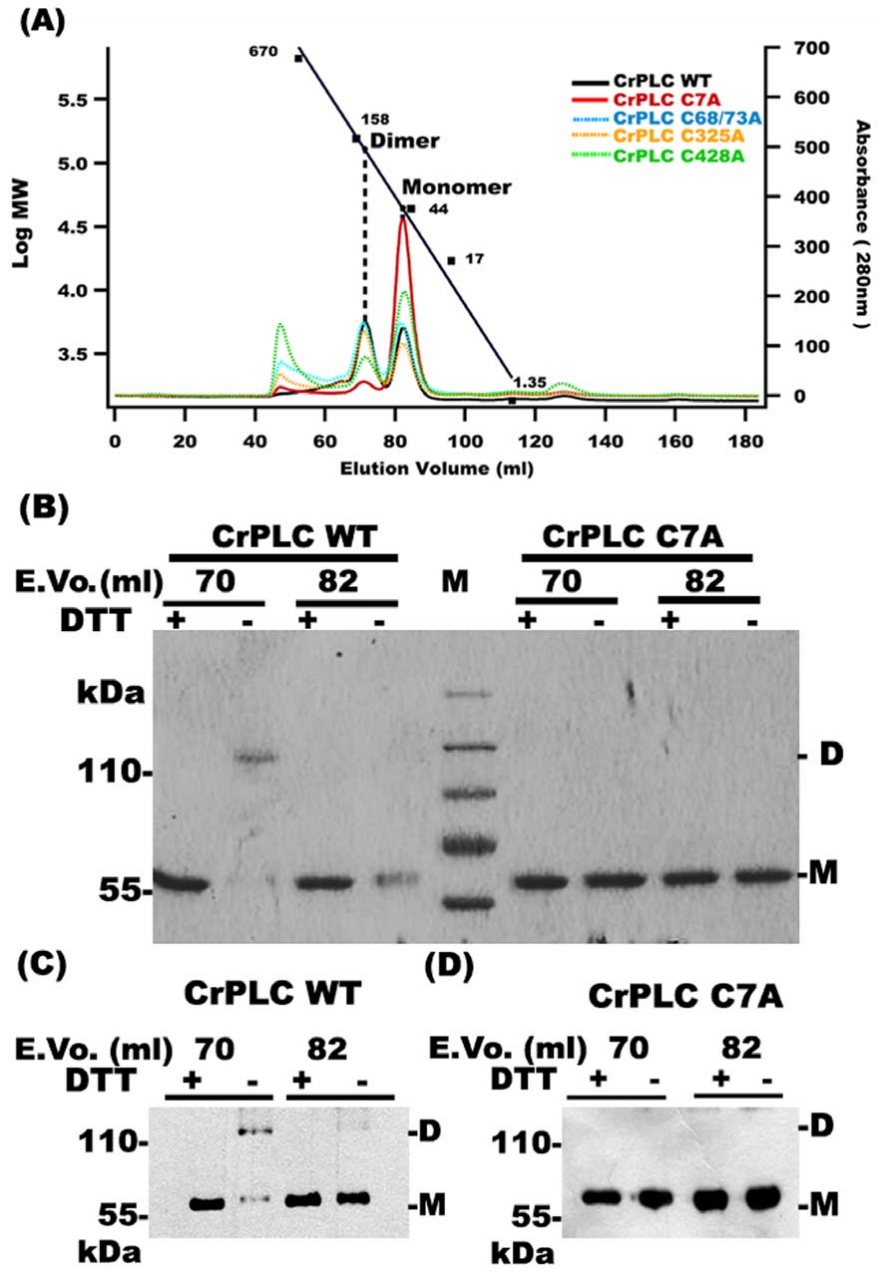
Cys7 plays critical role in CrPLC dimer stabilization. (A) Chromatogram of different CrPLC mutants indicated in different colors, overlaid with wild type CrPLC (CrPLC WT) shown in black. (B) SDS-PAGE analysis of eluents obtained at 70 ml and 82 ml with preparatory size exclusion chromatography of CrPLC WT and CrPLC C7A mutant under reducing (+DTT) and non-reducing (−DTT) conditions, respectively. (C and D) Immunoblot analysis of CrPLC WT and CrPLC C7A mutant protein respectively, under reducing (+DTT) and non-reducing (−DTT) conditions. Lanes are labeled by elution volume of monomer and dimer peak fractions obtained by preparatory size exclusion chromatography. M denotes the marker.

Disappearance of CrPLC dimer under reducing conditions, both *in vitro* and *in vivo* and the presence of eight cysteine residues in CrPLC protein sequence provided strong basis for inferring the involvement of disulphide bridge(s) in CrPLC dimerization. Moreover, bioinformatics analysis also suggested the existence of Cys7 residue in a bonded state. Hence, to investigate cysteine residues involved in the formation of disulphide bond in CrPLC, the most exposed cysteine residues, present at the N-terminal sequence (C7), catalytic domains (C68, C73) as well as C2 domain (C325 and C428) were mutated to alanine. The chromatographic data representing the elution profile of all CrPLC mutants were overlaid with the wild type (Figure 4A). As presented earlier, wild type CrPLC eluted as two major peaks of approximately equal ratio. In case of C7A mutant, the peak of dimer was found to be dramatically reduced and CrPLC C7A eluted as a monomer. SEC profile of C325A and C428A CrPLC were found to be similar to that of wild type protein. C68A and C73A double mutant formed higher oligomers with relatively less dimeric form. Immunoblotting of the corresponding elution fractions of CrPLC C7A mutant when compared with that of wild type and other mutants, showed a single band corresponding to monomer (Figure 4B; Figures S5A and B). The elution fractions of other CrPLC mutants including C68/73A, C325A and C428A have not shown considerable effect on dimerization (Figures S5C–E). These findings implied the importance of Cys7 mediated disulphide bond in the dimerization of CrPLC.

### Cys7 Mutation in CrPLC also Affects Lipid Affinity

**Figure 5:**
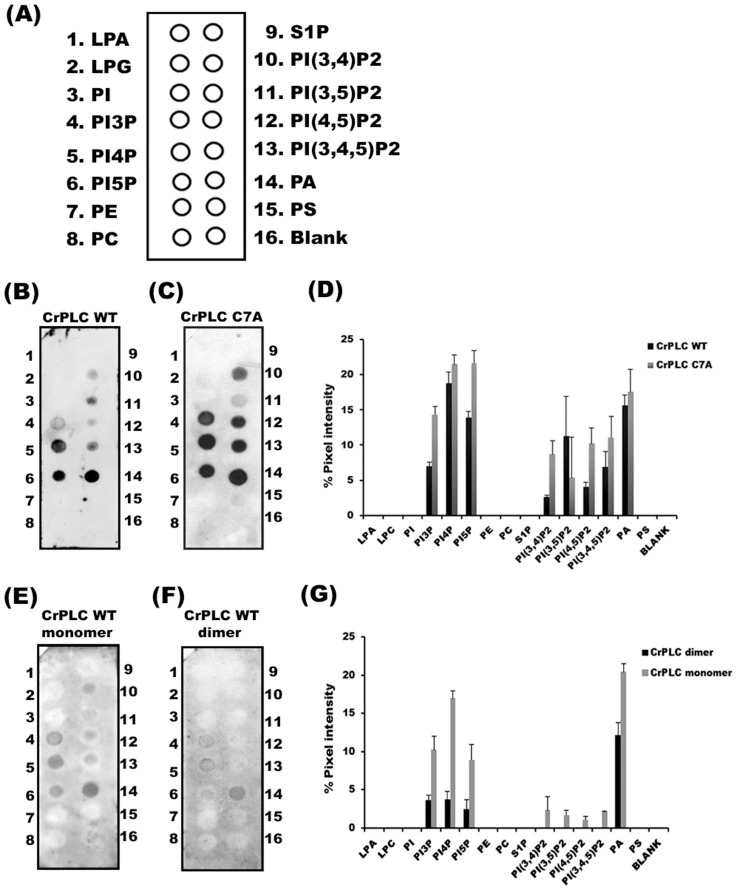
Interaction and affinity of CrPLC with phospholipids. (A) Schematic representation of PIP strip indicating the phospholipid present in each dot numbered from 1–16. Nomenclature of different phospholipids spotted on membrane are Lysophosphatidic acid (LPA), lysophosphatidylcholine (LPC), phosphatidylinositol (PtdIns), PtdIns(3)P, PtdIns(4)P, PtdIns(5)P, phosphatidylethanolamine (PE), 8-phosphatidylcholine (PC), sphingosine 1-phosphate, PtdIns(3,4)P2, PtdIns(3,5)P2, PtdIns(4,5)P2, PtdIns(3,4,5)P3, phosphatidic acid (PA), phosphatidylserine (PS), blank. (B and C) Lipid overlay using affinity purified CrPLC WT and CrPLC C7A mutant respectively. (D) Bar graph representing the densitometric measurement of binding of CrPLC WT and C7A mutant (E and F) Comparison of lipid binding affinity of the SEC purified monomer and dimer fractions, respectively. (G) Bar graph representing the densitometric measurement of binding of monomer and dimer fractions of SEC purified wild type CrPLC. Results represent the mean ± standard deviation of three independent experiments.

It has been proposed for few PI-PLC, that dimerization on the membrane surface could affect the affinity for its phospholipid substrate [27]. Similarly, the existence of CrPLC in dimerized form in *Chlamydomonas* could have implications for its interaction with phospholipid substrates. In order to characterize the functional significance of monomer and dimer species of CrPLC, lipid interaction of affinity purified recombinant CrPLC (wild type and mutants) and SEC purified monomer and dimer fractions were performed by lipid overlay experiments [15], [28], respectively. CrPLC WT that possess both monomer and dimer form showed association with a set of phospholipids (PIP) such as PI3P, PI4P, PI5P, PI(3,4)P2, PI(3,5) P2, PI94,5)P2, PI(3,4,5)P2 and PA with varying binding affinities (Figure 5B). Interestingly, CrPLC C7A mutant, which is mainly a monomer showed comparatively higher binding affinity with a set of PIPs when compared with the wild type CrPLC (Figures 5B and C). Since, C7A mutant showed significantly increased affinity with PIPs as compared with that of wild type CrPLC (Figure 5D), binding affinity of the dimer and monomer fractions of the wild type CrPLC were also compared (Figures 5E and F). Wild type CrPLC monomer showed binding with the same set of PIPs but with the much lower affinity (Figures 5E and G). The dimer fraction of WT CrPLC showed poorest lipid binding affinity (Figures 5F and G). These results suggested that the C7A mutant monomeric form potentiate the binding affinity of CrPLC to a set of PIPs. These results have left no doubt that the formation of disulphide bond and/or dimerization poses steric hinderance to some residues and prevents them from interacting with the set of PIPs. These results further implied that the process of dimerization might sterically hinder positively charged amino acid residues from interacting with negatively charged phospholipids head groups and hence the dimerization was found to possess lower lipid affinity and mutation in Cys7 residue which disrupted the dimer, had the highest lipid affinity.

### CrPLC Mainly Localizes to the Plasma Membrane in *C.* *reinhardtii*

**Figure 6:**
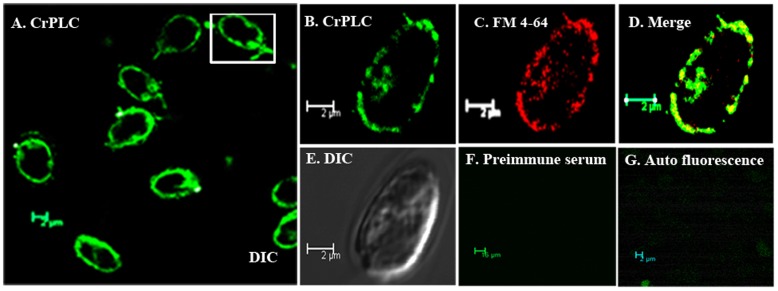
Cellular localization of CrPLC. (A) CrPLC localizes in the plasma membrane of *C. reinhardtii* cell as shown by green channel (B) Magnified image of a single cell. (C) Plasma membrane traced by using FM4-64 tracker dye is shown in red. (D) Merged image represent the overlay, where green and red channels are merged together to confirm CrPLC localization in the plasma membrane. (E) Cells visualized in DIC mode of light microscopy. (F and G) Immunolocalization with pre-immune serum and auto-fluorescence served as negative control.

Phospholipase has been proposed to function at the plasma membrane for regulation of different signaling cascades [29], [30]. Overall, cellular localization of CrPLC in *C. reinhardtii* cell displayed the preferential localization of CrPLC to the plasma membrane (Figures 6A and B). Labeling of the cells with the membrane tracer FM 4-64 (Figure 6C) suggested the co-localization of CrPLC with the plasma membrane and sub-cellular membrane vesicle compartment**.** This preferential localization might be due to high lipid affinity of CrPLC. Co-localization of CrPLC with the membrane of sub-cellular compartment does not rule out the possibility of translocation of CrPLC protein via sub-cellular membrane vesicle compartments to the plasma membrane. The co-localization signal of CrPLC with the membrane periphery and absence of such signal with preimmune serum or in autofluorescence images, confirmed its association mainly with the plasma membrane (Figures 6D–G). Membrane association of CrPLC could be essential for *C. reinhardtii* cell as it provides its access and availability to interacting partners. It is required to elucidate the functional importance of cellular localization of CrPLC after which the importance of the localization in the plasma membrane may become more obvious.

### Secondary Structure and Conformation Analysis of CrPLC and its Variants

**Figure 7:**
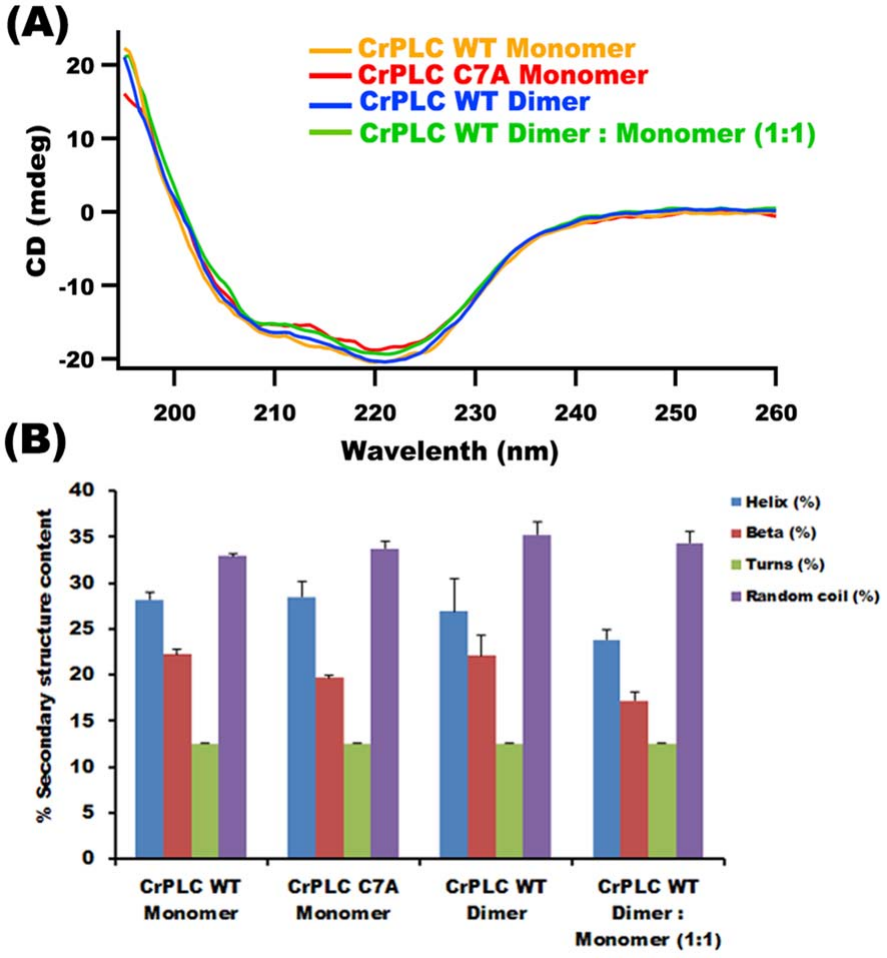
Secondary structure analysis of CrPLC. (A) Comparision of Far UV-CD spectra recorded for dimer and monomer fractions of CrPLC WT, CrPLC C7A mutant and equimolar fractions of mixed dimer and monomer CrPLC represented in different line patterns as indicated in inset. (B) Bar graph represent the calculated percentage secondary structure content including α-helix, β-sheets, turns and random coil content in CrPLC WT (monomer, dimer and dimer : monomer 1∶1) and CrPLC C7A mutant.

Secondary structure of the CrPLC WT (WT monomer, WT dimer and WT monomer and dimer mixture; 1∶1) and C7A mutant were experimentally determined by CD spectroscopy. Three independent far-UV CD spectroscopy experiments were performed. CD spectra of variants of CrPLC WT and C7A mutant between 190 and 260 nm showed strong minimum near 222 nm. CD spectra of CrPLC WT monomer, CrPLC WT dimer and CrPLC C7A mutant were compared. All the proteins were well folded and their secondary structure content including percentage alpha-helicity, turns and random coil content remained largely unchanged (Figures 7A and B **).** Quantification of secondary structure content of CrPLC variants were performed using Raussean et al [31] method, which utilizes experimental data points of the CD spectra to estimate secondary structure, such analysis showed comparable percentage of secondary structure content with that of predicted alpha helical component from the calculated tertiary structure model of CrPLC. Moreover, results obtained suggested that there is no significant change in the alpha helical content of the WT monomer (28.2%), WT dimer (26.9%) and monomer of C7A mutant (28.5%). There was a significant change in the content of beta sheet of the C7A mutant (19.7%) compared to that of WT monomer (22.2%). This difference might account for the minor conformational changes that may impact binding affinity of CrPLC to the set of PIPs.

### Conclusions

Disulphide bridges are known to play important roles in maintaining the structure of proteins and might regulate function of the proteins. This study provides strong evidences for the existence of a novel phospholipase from *C. reinhardtii* (CrPLC) as both monomer and dimer forms under physiological and *in vitro* conditions. In both the conditions, the disulphide linkages were found to promote dimerization. Our findings showed the importance of disulphide bridges in CrPLC dimerization. Three single mutants (C7A, C325A and C428A) and one double mutant C68/73A were generated for studying the role of cysteine in oligomerization and lipid interactions. Mutation C7A was mainly found to disrupt the dimerization in CrPLC whereas other mutants like C325A, C428A or C68/73A were not found to affect the CrPLC dimer but leading to the formation of higher oligomers of CrPLC protein. C7A mutation disrupted ∼95% of dimerization of CrPLC leading to the increase of monomer fraction of CrPLC by ∼2-fold and formation of small amount of higher oligomers. Thus, mutational analysis suggested the pivotal role of Cys7 in CrPLC dimer formation and stabilization. Moreover, substitution of Cys7 to alanine also altered the affinity of CrPLC for a set of phospholipids (substrates). Cys7 mutant potentiates the interaction of CrPLC towards a set of PIPs. However, monomer fraction of CrPLC wild type protein bound weakly and dimer fraction showed the poorest lipid interaction to the same set of PIPs. These experiments left no doubt that Cys7 mutation in CrPLC can potentiate the phospholipid interaction of CrPLC It would be interesting to carry out further studies to delineate the functional importance of the cellular localization of Cr.PLC in *Chlamydomonas.*

## Materials and Methods

### Bioinformatic Analysis

Putative CrPLC sequence of *C. reinhardtii* was fetched from its genome database (http://www.genome.jgi-psf.org/chlamy). Conserved domain architecture of CrPLC was analyzed by CDART program (http://www.ncbi.nlm.nih.gov/Structure/lexington/lexington.cgi) [32]. PLC sequences from different organisms were retrieved from NCBI and aligned using CLUSTALW multiple alignment tool (http://www.ebi.ac.uk/Tools/clustalw2/index.html ) for homology analysis [33]. Tertiary structure of CrPLC was predicted using homology modeling program (http://swissmodel.expasy.org/) and pdb files were viewed on PyMol. The predicted structure was verified using SAVES tool (http://nihserver.mbi.ucla.edu/SAVES/) and submitted to protein model database (PM0077883) [34]. Amino acid residues were located and highlighted in the tertiary structure model using PyMol program. Phylogenetic analysis was performed using MEGA version 5 [35]. Bonded Cysteines were predicted using CysPred server [36] and subcellular localization of CrPLC protein was predicted by Plant-mPLoc web-server [37].

### Heterologous Expression and Site Directed Mutagenesis of CrPLC

EST encoding CrPLC (accession number BP094257) was procured from Kajusa DNA research institute, Japan. A polynucleotide sequence that corresponds to CrPLC was PCR amplified with forward and reverse 5′-ATTGAATTCATGGGCAACGTGTTCAGCT-3′ and 5′-TTACTCGAGCTTGGGCCCGGCGTACAT-3′ primers respectively using EST as a template. Amplicon was cloned into pET21a (+) vector (Novagen, USA) that possess C-terminus hexa-histidine tag. Point mutations were created using the QuickChange mutagenesis kit (Stratagene, USA) and verified by automated DNA sequencing. CrPLC-pET21a was transformed into BL21 (DE3λ) *E. coli* cells. Cells were grown in terrific broth medium at 37°C and induced with 0.3 mM IPTG at cell culture O.D_600_ of 0.6. Protein expression was carried out at 16°C for 48 hrs. Recombinant proteins were purified by immobilized metal affinity chromatography using Co^2+^ metal ion resins (Clontech, Laboratories Inc. USA) according to manufacturer’s protocol.

### *Chlamydomonas* Culture and Cell Homogenate Preparation

*C. reinhardtii* strain cc 124 mt (−) used in this study was obtained from *Chlamydomonas* resource center (Department of Plant Biology, University of Minnesota). Culture was grown on TAP (tris-acetate phosphate media, pH 7.4 and supplemented with Hutners trace elements) at 25°C in an incubator shaker (120 rpm) with continuous exposure of fluorescent white light (2300 Lux). Cells in early exponential growth phase were used for all the experiments. *Chlamydomonas* total cell lysate (*Cr* TCL) was prepared by suspending cells in phosphate buffer saline (PBS; 150 mM NaCl, 10 mM sodium phosphate, and pH 7.2) in presence of protease inhibitor cocktail. Cell lysis was performed by sonication, giving 8 sec on and 8 sec off pulse for 6–8 times. Samples were solubilized by incubating crude lysate with 2×3% SDS Laemmli buffer for 30 min at 60°C.

### Size-exclusion Chromatography

Size exclusion chromatography was performed with a Akta explorer chromatography system (GE Healthcare USA) on HiLoad 16/60 Superdex 200 (1.0×30 cm) prep grade column and superose 12 10/300 GL analytical grade column, using FPLC mode, equilibrated with PBS, operated with flow rate of 1 ml/min at 4°C. Monomer and dimer species were determined by comparing it with standard molecular weight marker (Bio-Rad, USA), separated under the isocratic conditions. The peaks in the chromatogram were fit to a Gaussian curve with the IGOR Pro program (Wave Metrics Inc.’s).

### Western Blotting

Protein blotted nitrocellulose membrane was blocked using 5% non-fat dry milk powder in PBS supplemented with 0.1% tween-20 for 1 hr at room temperature. Blocked nitrocellulose membrane was then incubated with primary antibody generated against recombinant CrPLC at a 1∶3000 dilution. Immunolabelling was detected by using HRP (horseradish peroxidase) conjugated anti-rabbit secondary antibody (1∶5000 dilution) and visualized by enhanced chemiluminiscence method using the standard protocol.

### Glutaraldehyde Crosslinking

Protein crosslinking was performed by using 2.5% (v/v) freshly prepared glutaraldehyde (Sigma). 25 µM of heterologously expressed CrPLC or total cell protein of *C.* *reinhardtii* dissolved in 60 µl of PBS was incubated with 5 µl of 2.5% glutaraldehyde at 37°C. Reactions were stopped after 5 min by adding 1 M Tris-Cl, pH 8. Crosslinked protein samples were resolved on 10% SDS-PAGE and detected by immunoblotting.

### Immunolocalization of CrPLC in *Chlamydomonas*

Cells in early log phase were harvested and resuspended in PBS. Aliquots of approximately 200 µl of cell suspension were seeded on acid washed coverslips and fixed with 3.7% paraformaldehyde in PBS. Algal cells on the slide were permeabilized by submerging them in cold 100% ethanol at −20°C for 10 min. Cells were then washed with PBS containing 0.25 M NaCl at room temperature for 10 min, followed by PBS for 5 min. After brief washings with PBS containing 0.5% triton X-100 (PBST), incubation with freshly diluted rabbit CrPLC antiserum (1∶125 dilutions) or preimmune serum was carried out overnight at 4°C. Samples were incubated with FITC conjugated anti-rabbit IgG secondary antibody. Followed by brief washings, coverslips were mounted on slides applying antifade reagent (Slow Fade Gold, Molecular Probes). Slides were visualized by Leica TCS SP5 confocal microscope at central instrument facility, University of Delhi South Campus. Plasma membrane was labeled by tracker dye FM 4-64 (Invitrogen) at concentration 5 µg/ml.

### Lipid Overlay Assay

Lipid overlay assay was conducted using commercial PIP strips membranes (Molecular probes) [15] with spotted blots of synthetic phospholipids. Membrane was blocked with 1% skimmed milk in PBS buffer supplemented with 0.1% tween-20 for 1 hour at room temperature. The membrane was then incubated overnight on rocker with 2 µg/ml of recombinant CrPLC protein dissolved in PBST buffer, washed three times with PBST buffer for 10 min. Bound protein was then detected by standard immunoblotting as discussed earlier. Densitometry of blots was performed by Image J software (http://www.macbiophotonics.ca/imagej).

### CD Spectroscopy

Affinity purified CrPLC WT and C7A mutant were further purified by size exclusion chromatography against 10 mM Sodium Phosphate buffer pH 7.4. All the Far UV-CD measurements were performed in Jasco J815 spectrophotometer using quartz cuvette with the path length of 0.1 cm at room temperature. Spectra of each protein for protein concentration 0.2 mg/ml were recorded between 190–260 nm with the 1 nm band width and scan speed of 50 nm/min. Each spectrum was averaged over three scans and corrected by subtracting buffer baseline. The fractional α-helical, β-sheet, turns and random coil content of CrPLC WT and mutant protein was calculated using Rausseans et al method [31].

## Supporting Information

Figure S1 **Phylogenetic analysis of CrPLC.** Phylogenetic analysis comparing *C. reinhardtii* phospholipase C (CrPLC) with other PLC isoforms including sequences (accession numbers are given in brackets): *Homo sapiens* (AAH10668.2), *Mus Musculus* (CAM22088.1), *Catfish* (AAA87954.1), *Zea Mays* (ACG25330.1), *Oryza sativa* (ABA98951.2), *Nicotiana tabacum* (ABP57375.1), *Drosophila melanogaster* (ACZ95198.1), *Arabidopsis thaliana* (AAN75042.1), *Ostreococcus lucimarinus* (XP_003080695.1). The number at each branch point represents the bootstrap probability.
(TIF)

Figure S2 **Predicted tertiary structure of wild type CrPLC.** (A) Cartoon representation of the 3D structure of the CrPLC modeled using automated Swiss model server. (B) Position of aromatic amino acid and cysteine residues present on the surface of the molecule are shown in blue and red respectively.
(TIF)

Figure S3 **SDS-PAGE analysis of recombinant CrPLC.** Purification profile of the recombinant CrPLC purified by immobilized metal affinity chromatography and resolved on 10% SDS-PAGE.
(TIF)

Figure S4 **Immunoblot analysis of dimer and monomer fractions of CrPLC.** Separated monomer and dimer fractions purified by size exclusion chromatography corresponding to elution volume 67–76 ml and 79–88 ml respectively immunoblotted with CrPLC specific antibody.
(TIF)

Figure S5 **Immunoblot analysis of monomer and dimer fractions of wild type and various mutants of CrPLC under reducing and non-reducing conditions.** (A–E) Immunoblotting of both dimer (right panel) and monomer elution fractions (left panel) of CrPLC wild type and mutants under reducing (+DTT) and non-reducing (−DTT) conditions with CrPLC specific antibody.
(TIF)
