# Supplementary figures and images for: Disulphide Bridges of Phospholipase C of Chlamydomonas reinhardtii Modulates Lipid Interaction and Dimer Stability
Source: PLoS One. 2012 Jun 21;7(6):e39258. doi: 10.1371/journal.pone.0039258 (PMC3380823; doi:10.1371/journal.pone.0039258)

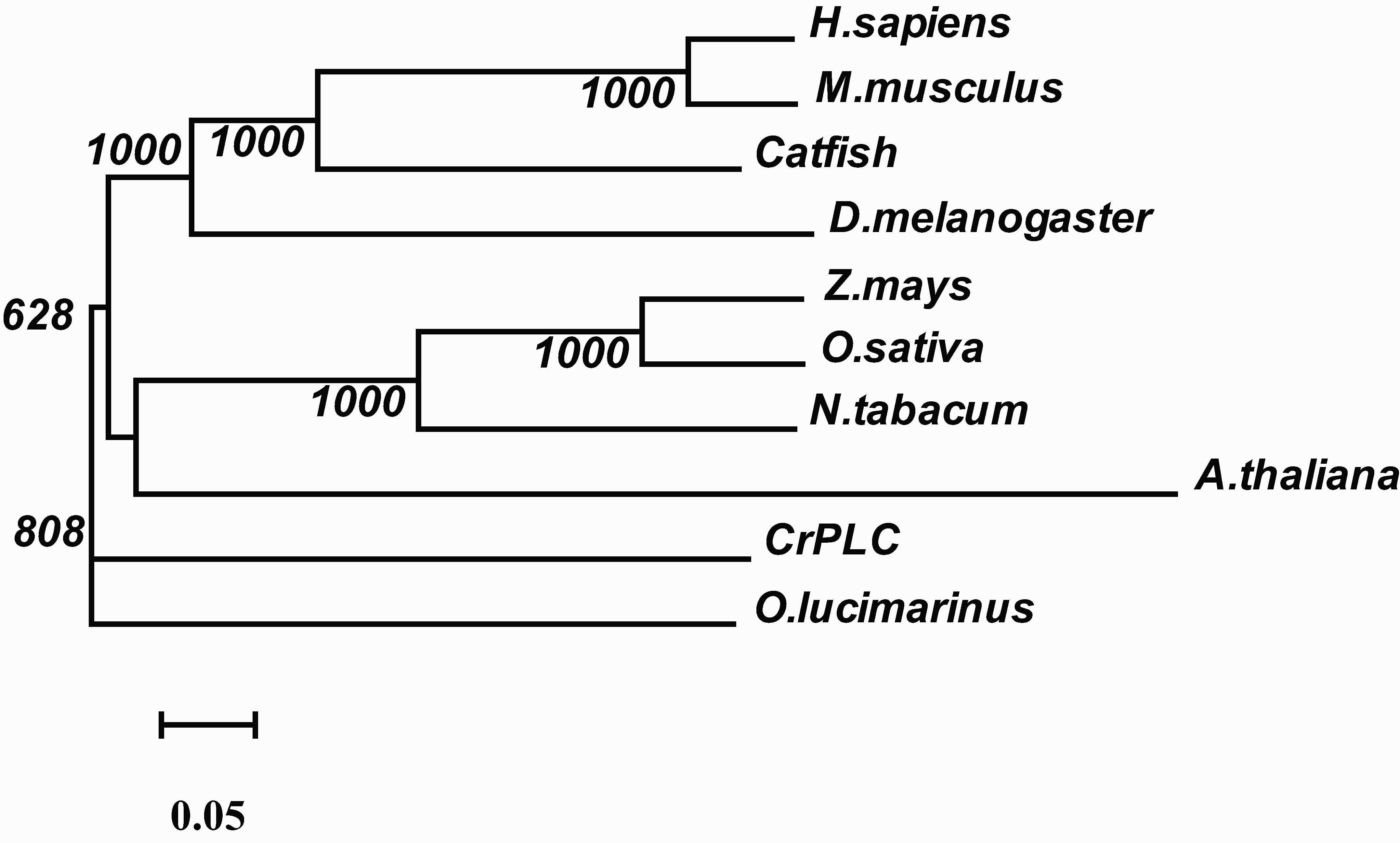

Supplement: Figure S1 — Phylogenetic analysis of CrPLC. Phylogenetic analysis comparing C. reinhardtii phospholipase C (CrPLC) with other PLC isoforms including sequences (accession numbers are given in brackets): Homo sapiens (AAH10668.2), Mus Musculus (CAM22088.1), Catfish (AAA87954.1), Zea Mays (ACG25330.1), Oryza sativa (ABA98951.2), Nicotiana tabacum (ABP57375.1), Drosophila melanogaster (ACZ95198.1), Arabidopsis thaliana (AAN75042.1), Ostreococcus lucimarinus (XP_003080695.1). The number at each branch point represents the bootstrap probability. (TIF) [file pone.0039258.s001.tif]

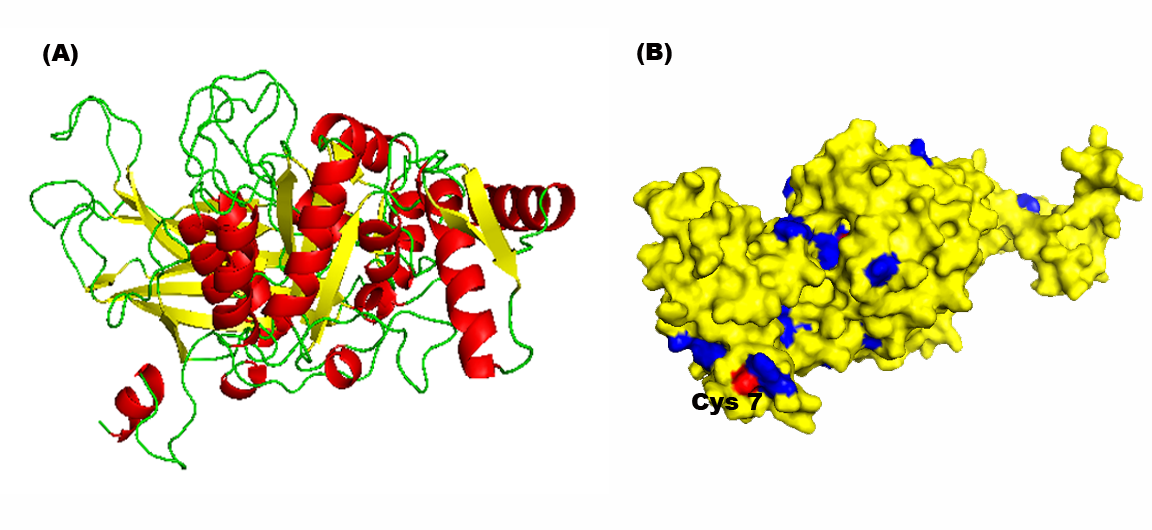

Supplement: Figure S2 — Predicted tertiary structure of wild type CrPLC. (A) Cartoon representation of the 3D structure of the CrPLC modeled using automated Swiss model server. (B) Position of aromatic amino acid and cysteine residues present on the surface of the molecule are shown in blue and red respectively. (TIF) [file pone.0039258.s002.tif]

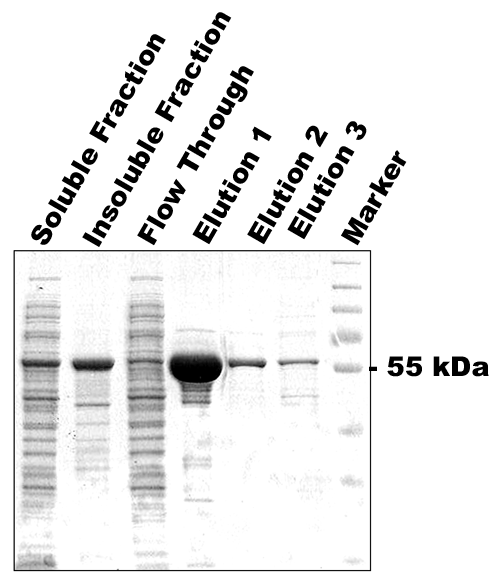

Supplement: Figure S3 — SDS-PAGE analysis of recombinant CrPLC. Purification profile of the recombinant CrPLC purified by immobilized metal affinity chromatography and resolved on 10% SDS-PAGE. (TIF) [file pone.0039258.s003.tif]

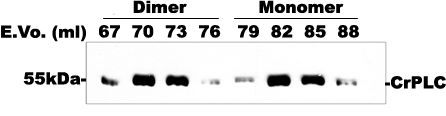

Supplement: Figure S4 — Immunoblot analysis of dimer and monomer fractions of CrPLC. Separated monomer and dimer fractions purified by size exclusion chromatography corresponding to elution volume 67–76 ml and 79–88 ml respectively immunoblotted with CrPLC specific antibody. (TIF) [file pone.0039258.s004.tif]

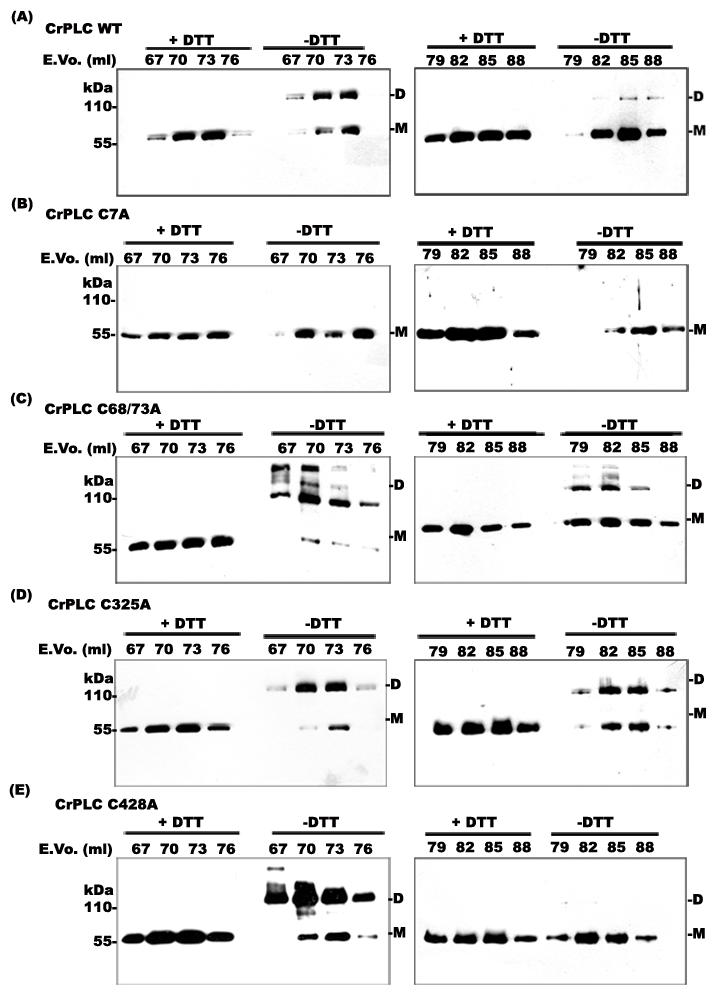

Supplement: Figure S5 — Immunoblot analysis of monomer and dimer fractions of wild type and various mutants of CrPLC under reducing and non-reducing conditions. (A–E) Immunoblotting of both dimer (right panel) and monomer elution fractions (left panel) of CrPLC wild type and mutants under reducing (+DTT) and non-reducing (−DTT) conditions with CrPLC specific antibody. (TIF) [file pone.0039258.s005.tif]
